# 

**Published:** 1829-01

**Authors:** 


### TO READERS AND CORRESPONDENTS.

The Editor is happy to find, that he has at length completed the publication of a number of the Journal, within the month for which it was designed, and confidently expects to accomplish the same thing with every future number.

The next number will contain a copious digest of Intelligence, both foreign and domestic.

To his correspondents, the Editor would remark, that such is the increasing patronage extended to the Journal, that whatever they write, will be read by a number sufficiently great to justify the most ambitious. In two months more, a new volume will commence, which with their aid he hopes will prove superior to the present. Such of them, as feel disposed to inscribe themselves in its first numbers, would do well to turn their attention forthwith to the preparation of papers.

The Editor was in hopes to have given in this number, the proceedings of the first meeting of the State Medical Society of Ohio, held at Columbus within the present month, but has not yet been able to obtain the requisite information.

